# Nurses' perspectives on privacy and ethical concerns regarding artificial intelligence adoption in healthcare

**DOI:** 10.1016/j.heliyon.2024.e36702

**Published:** 2024-08-22

**Authors:** Moustaq Karim Khan Rony, Sharker Md. Numan, Khadiza Akter, Hasanuzzaman Tushar, Mitun Debnath, Fateha tuj Johra, Fazila Akter, Sujit Mondal, Mousumi Das, Muhammad Join Uddin, Jeni Begum, Mst. Rina Parvin

**Affiliations:** a Master of Public Health, Bangladesh Open University, Gazipur, Bangladesh; b School of Science and Technology, Bangladesh Open University, Gazipur, Bangladesh; c Master of Public Health, Daffodil International University, Dhaka, Bangladesh; d Department of Business Administration, International University of Business Agriculture and Technology, Dhaka, Bangladesh; e Master of Public Health, National Institute of Preventive and Social Medicine, Dhaka, Bangladesh; f Masters in Disaster Management, University of Dhaka, Dhaka, Bangladesh; g Dhaka Nursing College, Affiliated with the University of Dhaka, Bangladesh; h Master of Science in Nursing, National Institute of Advanced Nursing Education and Research Mugda, Dhaka, Bangladesh; i Master of Public Health, Leading University, Sylhet, Bangladesh; j Master of Public Health, RTM Al-Kabir Technical University, Sylhet, Bangladesh; k School of Medical Sciences, Shahjalal University of Science and Technology, Bangladesh; l Bangladesh Army (AFNS Officer), Combined Military Hospital, Dhaka, Bangladesh

**Keywords:** Artificial intelligence, Healthcare, Privacy, Ethical concerns, Nurses

## Abstract

**Background:**

With the increasing integration of artificial intelligence (AI) technologies into healthcare systems, there is a growing emphasis on privacy and ethical considerations. Nurses, as frontline healthcare professionals, are pivotal in-patient care and offer valuable insights into the ethical implications of AI adoption.

**Objectives:**

This study aimed to explore nurses' perspectives on privacy and ethical concerns associated with the implementation of AI in healthcare settings.

**Methods:**

We employed Van Manen's hermeneutic phenomenology as the qualitative research approach. Data were collected through purposive sampling from the December 7, 2023 to the January 15, 2024, with interviews conducted in Bengali. Thematic analysis was utilized following member checking and an audit trail.

**Results:**

Six themes emerged from the research findings: Ethical dimensions of AI integration, highlighting complexities in incorporating AI ethically; Privacy challenges in healthcare AI, revealing concerns about data security and confidentiality; Balancing innovation and ethical practice, indicating a need to reconcile technological advancements with ethical considerations; Human touch vs. technological progress, underscoring tensions between automation and personalized care; Patient-centered care in the AI era, emphasizing the importance of maintaining focus on patients amidst technological advancements; and Ethical preparedness and education, suggesting a need for enhanced training and education on ethical AI use in healthcare.

**Conclusions:**

The findings underscore the importance of addressing privacy and ethical concerns in AI healthcare development. Nurses advocate for patient-centered approaches and collaborate with policymakers and tech developers to ensure responsible AI adoption. Further research is imperative for mitigating ethical challenges and promoting ethical AI in healthcare practice.

## Highlights

• Nurses likened using AI in patient care to storytelling, emphasizing ethical decision-making akin to crafting a narrative.
• Participants emphasized their role as compassionate healthcare guardians, prioritizing patient privacy and ethical guidelines amid technological advancements.
• Nurses highlighted their crucial responsibility in AI adoption, ensuring ethical technology use and safeguarding patient data.
• Nurses expressed data security concerns, seeing themselves as patient information guardians.
• Nurses emphasized tech-ethics, empowering patients with responsible AI, while ensuring compassionate, personalized care.

## 1 Introduction

In the contemporary landscape of healthcare, the integration of artificial intelligence (AI) has emerged as a transformative force, promising enhanced efficiency, accuracy, and patient outcomes [1]. However, alongside its potential benefits, the adoption of AI technologies in healthcare also raises significant ethical and privacy concerns [2]. Nurses, as frontline healthcare professionals, play a pivotal role in the implementation and utilization of AI systems within clinical settings [3]. Their perspectives on the ethical implications and privacy considerations associated with AI adoption are therefore crucial for understanding the broader implications of this technological advancement [4]. Artificial intelligence encompasses a diverse array of technologies, ranging from machine learning algorithms to natural language processing systems, all designed to analyze vast amounts of data and derive actionable insights [5,6]. AI systems are built to simulate human intelligence processes, such as learning, reasoning, and problem-solving, with the aim of performing tasks that traditionally require human intelligence [7].

In healthcare, AI holds the promise of revolutionizing various aspects of clinical practice, including diagnosis, treatment planning, and patient monitoring [8]. By harnessing the power of AI, healthcare providers can potentially improve diagnostic accuracy, optimize treatment protocols, and personalize care delivery to individual patients' needs [9]. Nurses, in their clinical roles, experience the direct impact of AI technologies. For instance, AI-powered diagnostic tools can assist nurses in interpreting complex medical data more efficiently, enabling timely interventions and improving patient outcomes [10]. Similarly, AI-driven predictive analytics can help nurses identify patients at high risk of deterioration, allowing for proactive interventions and resource allocation [11]. Additionally, natural language processing systems can facilitate smoother communication and documentation processes, streamlining workflows and enhancing patient care coordination [12].

Despite its transformative potential, the widespread adoption of AI in healthcare is not without its challenges [13]. One of the foremost concerns relates to the ethical implications of AI-driven decision-making in clinical practice [14]. As AI algorithms increasingly assist healthcare providers in making diagnostic and treatment decisions [15], questions arise regarding the transparency, accountability, and fairness of these algorithms [16]. Nurses, who often collaborate closely with AI systems in their daily practice, are uniquely positioned to offer insights into the ethical dilemmas [17] that arise when human judgment is augmented or replaced by AI-driven processes [18]. Moreover, the integration of AI technologies into healthcare raises profound privacy concerns, particularly concerning the security and confidentiality of patient data [19]. AI systems rely on access to large datasets to train and refine their algorithms, raising questions about data privacy, consent [20], and the potential for unauthorized access or misuse of sensitive health information [21]. Nurses, who are entrusted with safeguarding patient confidentiality and upholding ethical standards of care, are acutely aware of the implications of data privacy breaches and the importance of maintaining patient trust in the era of AI-enabled healthcare [22].

Furthermore, the ethical and privacy concerns surrounding AI adoption intersect with broader issues of social justice, equity, and access to healthcare [23]. As AI algorithms rely on historical data to generate predictions and recommendations, there is a risk of perpetuating biases and disparities present in the healthcare system [24]. These biases can manifest in various ways, such as unequal treatment recommendations or misdiagnoses based on flawed data sets, leading to ethical dilemmas in patient care [25]. Nurses, who are advocates for patient welfare and social justice, are attuned to the potential implications of AI technologies for vulnerable and marginalized populations, including issues of algorithmic bias, discrimination, and inequitable access to care [26]. Ethical issues also encompass patient consent, transparency in AI decision-making processes, and the safeguarding of patient data against misuse or breaches [27]. In light of these complex ethical and privacy considerations, it is imperative to engage nurses in discussions about the responsible development, implementation, and oversight of AI technologies in healthcare [28]. Nurses' perspectives can provide valuable insights into the practical challenges and ethical dilemmas encountered in the frontline implementation of AI systems [29], as well as inform the development of policies and guidelines to ensure the ethical and responsible use of AI in clinical practice [30].

Previous studies recommended elucidating the nuanced ethical dilemmas and privacy considerations that arise from the integration of AI technologies into healthcare [[31], [32], [33], [34]]. Therefore, drawing on empirical research and qualitative interviews with nurses working in diverse clinical settings, this research aimed to explore nurses' perspectives on privacy and ethical concerns regarding the adoption of artificial intelligence in healthcare settings. By amplifying nurses' voices in the discourse surrounding AI ethics, we hope to foster greater awareness, dialogue, and collaboration towards the development of ethically sound and socially responsible AI-driven healthcare systems.

## 2 Research question

What are nurses' perspectives on privacy and ethical concerns regarding artificial intelligence adoption in healthcare?

## 3 Methods

### 3.1 Research design

In this study, we employed Van Manen's hermeneutic phenomenology as the qualitative research approach [35,36] to delve into nurses' viewpoints concerning privacy and ethical considerations tied to the integration of Artificial Intelligence (AI) in healthcare. The choice of hermeneutic phenomenology was driven by the recognition of the intricate and subjective nature of the phenomena under investigation [37,38]. Employing Van Manen's hermeneutic phenomenology within thematic analysis allowed for a nuanced exploration of the interplay between individual experiences and broader contextual factors, ensuring a holistic understanding of nurses' viewpoints [[39], [40], [41]]. This approach facilitated the identification of key themes while capturing the depth and complexity of participants' narratives, contributing to a robust and insightful analysis [42]. By focusing on nurses' interactions with AI adoption in healthcare, this approach uncovered the underlying meanings embedded in their experiences, providing a thorough examination of the subjective perspectives on privacy and ethical considerations. Additionally, adherence to the Consolidated Criteria for Reporting Qualitative Research (COREQ) checklist ensured methodological rigor and transparency throughout the study, enhancing the credibility and trustworthiness of the findings [43].

### 3.2 Study setting

This study was conducted among respondents from the four largest tertiary hospitals situated in Dhaka city, Bangladesh. These hospitals were selected for their diverse patient populations and advanced healthcare services, providing an ideal environment to explore nurses' perspectives on privacy and ethical concerns related to the adoption of artificial intelligence in healthcare. Participants experienced various forms of artificial intelligence integration, such as AI-driven diagnostic tools, smart monitoring systems, and predictive analytics software, within their daily workflow.

Within these hospitals, the nursing system operates under a hierarchical structure, with registered nurses (RNs) overseeing patient care and delegating tasks to nursing assistants. Nurses typically work shifts lasting 6–12 h, often rotating to ensure continuous patient care. The average nurse-to-patient ratio in the study setting was approximately 1:9, which poses significant challenges in providing personalized care [44]. Patient-to-nurse ratios vary by unit and time of day, with critical care units maintaining lower ratios for more intensive monitoring, while general wards experience higher ratios.

### 3.3 Conceptual framework

**Fig. 1:**
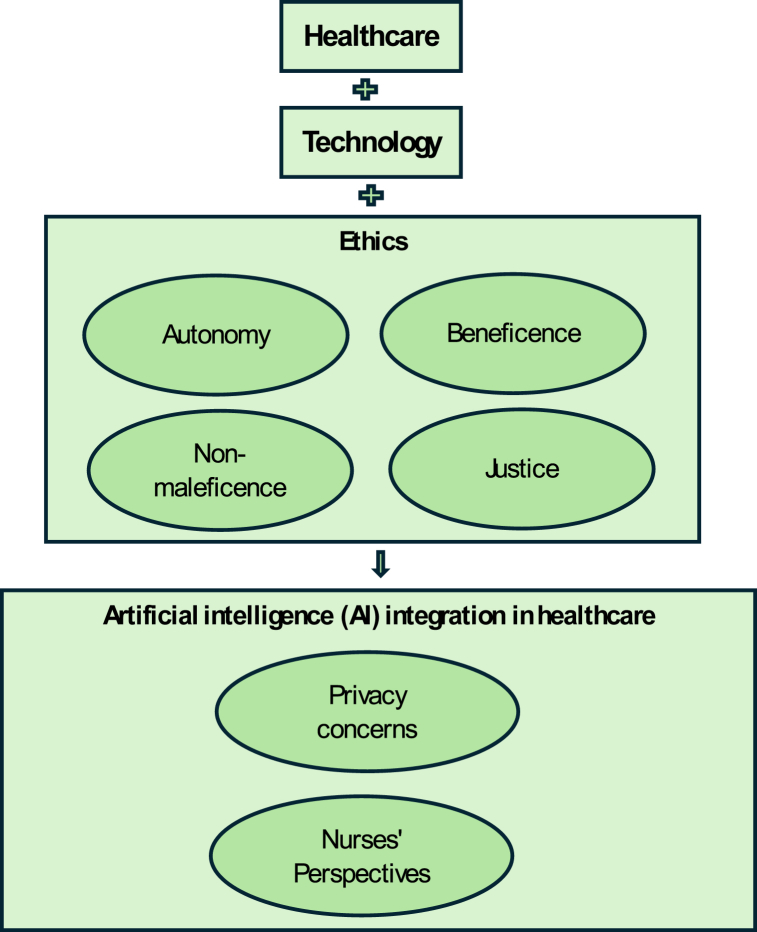
Conceptual framework.

In this research, the conceptual framework (Fig. 1) was grounded in the intersection of healthcare, technology, ethics, and human experience [[45], [46], [47], [48]]. Drawing from the ethical principles of autonomy, beneficence, non-maleficence, and justice [49,50], this study explored how nurses perceive the integration of artificial intelligence (AI) in healthcare, particularly concerning privacy and ethical considerations. Guided by the conceptual framework, the research delved into the moral implications of AI adoption, examining nurses' perspectives on the potential benefits of AI technologies, along with the imperative to safeguard patient privacy and uphold ethical standards. By employing semi-structured interviews and thematic analysis, this research illuminated the nuanced interplay between technology, ethics, and human values in the evolving landscape of healthcare.

### 3.4 Data collection

Data were collected using purposive sampling techniques [51] between December 7th, 2023, and January 15th, 2024. Individual semi-structured interviews were conducted to gather rich and detailed information about nurses' perspectives on privacy and ethical concerns related to AI in healthcare. The interview questions were designed through a meticulous process to ensure they aligned with the study's research objectives. Initially, a comprehensive set of questions was formulated based on studies related to AI and healthcare [[45], [46], [47], [48], [49], [50]]. The validity and reliability of the interview questions were confirmed through a series of steps. First, a pilot test involving five participants was conducted, and their feedback was used to revise the questions. Next, the questionnaire underwent review by two individual public health experts, and further refinement was made based on their feedback. The interview questions (Table 1) were asked in the Bangla language. The sample size (n = 20) was determined through data saturation, where recruitment continued until no new themes or insights emerged from the interviews [51]. Additionally, interviews were conducted with three participants to confirm whether data saturation had been achieved.

**Table 1:** Semi-structured interview questions.

How do you anticipate the integration of AI in healthcare settings will impact your role as a nurse, particularly in relation to concerns about patient privacy and ethics?
Looking into the future, what ethical considerations do you foresee arising with the increased adoption of AI in healthcare, and how might these concerns influence nursing practices?
In your opinion, what proactive measures can nurses take to address potential privacy issues as AI becomes more prevalent in healthcare, and how can ethical guidelines be shaped to accommodate these technological changes?
Considering the future landscape of healthcare with AI, how do you think the nurse-patient relationship might evolve, and what steps can be taken to uphold patient privacy and trust in this changing environment?
As AI becomes more integrated into healthcare, what kind of training and educational resources do you believe nurses will need to effectively navigate the ethical implications associated with these technological advancements?
Could you please share your thoughts on specific scenarios or contexts in which ethical dilemmas related to AI in healthcare may arise? Additionally, how would you propose addressing these challenges proactively?
With the ongoing adoption of AI in healthcare, what role do you see nurses playing in advocating for patient privacy and ethical considerations, and how can the nursing community contribute to the responsible implementation of AI tools in healthcare settings?

### 3.5 Participant selection

In this investigation, nurses meeting specific criteria were selected for participation. These criteria included holding a minimum master's degree, possessing a current nursing practice license, and being employed at the chosen tertiary-level hospital. The decision to focus on nurses with a minimum master's degree stemmed from the aim to gather insights from highly qualified healthcare professionals committed to continuous professional development. This study was conducted in two phases. In Phase 1, six participants were purposefully chosen through a social media circular (WhatsApp and Facebook groups) from among those working in the selected tertiary-level hospital in Dhaka city, as we obtained a shortlist of nurses from the study settings. The selection process also aimed to identify participants with the highest educational background and experience among those who met the inclusion criteria. Subsequently, in Phase 2, fourteen participants were purposively selected from the partial list of nurses. In total, twenty participants were included in the study.

### 3.6 Data management

The interviews were conducted by three experienced researchers trained in qualitative interviewing techniques, ensuring consistency and adherence to the interview guidelines. Participants selected for Phases 1 & 2 were contacted via email or phone to schedule a 50–60-min online interview through Zoom, with one researcher facilitating the interview while the other two researchers served as the third observer to ensure thorough data capture and adherence to interview procedures. A single interview was conducted with each participant. All interviews were audio-recorded with participants' consent. After a thorough review of the recorded data, translation from Bangla to English was performed with the assistance of two bilingual experts proficient in both languages.

### 3.7 Data analysis

In this study, thematic analysis was conducted through three distinct phases: identifying essential themes, analyzing thematic structures, and interpreting lived experiences [42]. In the phase of identifying essential themes, three researchers independently engaged with the same set of interview transcripts to discern the core themes that emerged from the data, ensuring a comprehensive understanding of the participants' lived experiences. Their interpretations closely aligned, demonstrating a shared recognition of the key elements within the data, which underscores the depth and reliability of our findings. Next, in the analysis of thematic structures, the same researchers collaborated to synthesize these themes, identifying patterns and relationships that formed sub-themes. These sub-themes were meticulously refined and synthesized to reveal overarching main themes. Finally, in the phase of interpreting lived experiences, the researchers delved into these main themes to encapsulate the essence of nurses' perspectives on privacy and ethical concerns related to AI adoption in healthcare. This interpretive process facilitated a deep and nuanced understanding of the data, providing rich insight into the complex dimensions of nurses' viewpoints on the intersection of AI technology, patient privacy, and ethical considerations within the healthcare landscape.

### 3.8 Trustworthiness

To enhance the rigor and trustworthiness of the study, several measures were implemented. Firstly, member checking was conducted to validate the findings with participants, ensuring accuracy and authenticity [52]. This process involved utilizing datasets in the Bangla language. Additionally, to promote transparency and accountability in the methodology, an audit trail was carefully maintained throughout the research process [53]. The audit trail included detailed documentation of all research activities, from initial data collection through to analysis and interpretation. Each step, decision, and modification made during the research was recorded to ensure traceability and verifiability of the findings. Moreover, reflexivity was addressed by the researcher, who critically examined their own role, potential biases, and influence on the research. This introspective approach aimed to acknowledge and mitigate any subjective interpretations or preconceptions [54]. Furthermore, the researchers' experience and training were disclosed, providing insight into their qualifications and perspectives. Lastly, reflexive bracketing was employed to foster openness and reflexivity, encouraging the researchers to bracket their preconceived notions and maintain an open-minded stance throughout the research process [55]. These methodological strategies collectively contribute to the rigor and trustworthiness of the study, ensuring robustness in data collection, analysis, and interpretation.

### 3.9 Ethical considerations

This study obtained ethical clearance from the review committee of Bangladesh Open University, Bangladesh, with approval number BOU/MPH/HR08/11112023. Informed consent was obtained from all participants before their involvement in the study. Participants were provided with information about the research purpose, procedures, potential risks, and their right to withdraw at any time without consequences. Additionally, participants' confidentiality was rigorously maintained by assigning pseudonyms and removing any identifiable information from transcripts. The dataset was securely stored, accessible only to the research team. Transcripts were also stored separately from personal information to further safeguard participants' privacy.

## 4 Results

### 4.1 Participants' characteristics

Twenty respondents were involved in that study (Table 2), with ages ranging from 28 to 52 years (Mean = 37.2 years; SD = 6.47). The majority of the participants were female (n = 12; 60 %) and male (n = 8; 40 %). Additionally, participants' work experience ranged from 3 to 27 years (Mean = 13.2 years; SD = 6.95). The majority of the nurses held a master's degree (n = 18; 90 %), with a smaller number possessing a Doctor of Nursing Practice (n = 2; 10 %).

**Table 2:** Respondents' demographic profile.

**Respondents' code**	**Gender**	**Age**	**Designation**	**Highest education**	**Work experience (years)**	**Working area**
R01	Female	39	Nursing incharge	Master of Public Health	15	Emergency
R02	Male	28	Senior staff nurse	Masters in clinical social work	4	Coronary care unit
R03	Female	46	Nursing manager	Doctor of Nursing Practice	22	Intensive care unit
R04	Female	41	Nursing incharge	Master of Public Health	17	Coronary care unit
R05	Male	37	Senior staff nurse	Master of Public Health	13	Emergency
R06	Male	30	Senior staff nurse	Masters in nursing	6	Emergency
R07	Female	35.5	Senior staff nurse	Master of Public Health	11	Operation theater
R08	Female	43	Nursing incharge	Master of Public Health	19	Obstetrics and gynaecology
R09	Female	49	Nursing manager	Master of Public Health	25	Surgical unit
R10	Male	36	Senior staff nurse	Masters in nursing	21	Coronary care unit
R11	Female	40	Nursing incharge	Masters in Nutrition	16	Operation theater
R12	Female	28	Senior staff nurse	Master of Public Health	3	Emergency
R13	Male	32	Senior staff nurse	Masters in gerontology	7	Emergency
R14	Female	35	Senior staff nurse	Master of Public Health	9	Emergency
R15	Female	38	Nursing incharge	Masters in nursing	14	Obstetrics and gynaecology
R16	Female	52	Nursing manager	Doctor of Nursing Practice	27	Medicine unit
R17	Male	31	Senior staff nurse	Master of Public Health	5	Intensive care unit
R18	Male	33	Senior staff nurse	Master of Public Health	8	Emergency
R19	Female	38.5	Nursing incharge	Masters in nursing	15	Emergency
R20	Male	32	Senior staff nurse	Master of Public Health	7	Intensive care unit

### 4.2 Ethical dimensions of AI integration

#### 4.2.1 Informed decision-making

The findings of this study highlighted that employing AI for patient care goes beyond simple button presses; it's akin to crafting a narrative through choices (Table 3). Each decision, viewed as a chapter in a story, transcends mere task completion. Delving into abundant information, participants ensured their choices aren't hasty but a carefully devised plan, aligning with ethical principles. The process of making good decisions with AI in patient care resembles storytelling, where thoughtful considerations shape a tale of choices adhering to the values and rules they believe in—a narrative reflective of their commitment to responsible healthcare practices.

"When we use AI to help take care of patients, it's not just pressing buttons; it's like creating a story with our choices. Each decision is like a part of a story, not just finishing tasks. We look at a lot of information, making sure our choices aren't quick but a well-thought-out plan, following what's right. Making good decisions with AI in patient care is like telling a story where the ideas come from thinking carefully, making a tale of choices that follow the rules we believe in." *(Code: R13, Male, Senior staff nurse, 7 years of working experience)*

**Table 3:** Themes, sub-themes, and codes.

**Themes**	**Sub-themes**	**Codes**
Ethical dimensions of AI integration	Informed decision-making	AI, Patient care, Decision-making, Story, Choices, Information, Well-thought-out plan, Right, Good decisions, Rules, Thinking carefully, Tale
	Ethical guidelines and frameworks	Nurses, Healthcare, Rules, Right, AI, Patients' information, Respect, Values, Ethical guidelines, Trust, Technology, Progress, Ethical rules, Journey, Responsibility, Patient privacy, Principles, Caring, Honest, Special
	Professional accountability and responsibility	Nurses, New technology, AI, Healthcare, Responsibility, Patients' information, Right, Wrong, Decision-making, Change, Help, Problems, Mixing technology, Promises, Rules, Trust, Safety, Care, Well taken care of
Privacy challenges in healthcare AI	Data security and confidentiality	AI, Patient information, Privacy, Nurses, Trust, Responsibility, Technology, Computer program, Digital connection, Secret, Guardians, Safe healthcare future, Builders, Special connection, Safekeeping
	Algorithmic transparency and accountability	Nurses, People, Computer stuff, Secrets, Numbers, Transparency, Right thing, Healthcare, Computer help, Protectors, Private information, Wrong hands
	Patient advocacy for privacy	Nurses, Leaders, Play, Computers, Healthcare, Promise, Private things, Trust, Protection, Person, Help

#### 4.2.2 Ethical guidelines and frameworks

The findings of this study emphasized the nurses' role as compassionate guardians in healthcare, particularly when incorporating new technologies like AI. Their commitment extends to preserving patient privacy and maintaining respect. Despite the technological landscape, participants stressed the importance of upholding core values. Ethical rules were likened to protective walls, safeguarding the trust between nurses and those under their care. This qualitative insight underscored the dedicated adherence to ethical principles and the pivotal role they play in balancing technological advancements with fundamental values in healthcare.

"Nurses are like caring protectors in healthcare. They follow the rules and do what's right, especially when new things like AI are being used. We promise to keep patients' information private and treat them with respect. Even in a world of technology and information, we make sure that progress doesn't forget the important values of our job. Ethical rules are like walls that keep the trust between nurses and the people we take care of safe." *(Code: R05, Male, Senior staff nurse, 13 years of working experience)*

Participants stressed the importance of adhering to ethical guidelines when integrating AI into healthcare. The belief in reinforcing a sense of right and wrong with new technology emerged, emphasizing that ethical rules serve as guiding beacons rather than limitations. Ethical guidelines were depicted as bright signs illuminating the path to maintaining honesty, care, and fidelity to healthcare principles. The responsibility of using AI was seen as a commitment to respecting patient privacy and upholding the core values that make healthcare unique, providing valuable insights into the ethical considerations in AI adoption.

"I believe that as nurses using AI in healthcare, it's important to follow ethical guidelines. When we use new technology, we don't forget about what's right and wrong; instead, we make our sense of what's right even stronger. Ethical rules aren't rules that limit us; they're like bright signs showing us the way to keep our journey honest, caring, and true to the principles of our job. As we move forward with new ideas and ethics, we hold the responsibility of using AI in a way that respects patient privacy and follows the values that make healthcare special." *(Code: R19, Female, Nursing incharge, 15 years of working experience)*

#### 4.2.3 Professional accountability and responsibility

In this study, participants emphasized their crucial role in adopting new technologies like AI in healthcare. The responsibility of ensuring the right use of technology was highlighted, with a particular emphasis on safeguarding patient information. The participants stressed the need to carefully consider the ethical dimensions in every decision related to AI use, acknowledging the transformative nature of this change. Balancing the potential benefits for patients with the cautious management of potential issues underscored the complex role they play in navigating the integration of AI in healthcare.

"Nurses are very important when it comes to using new technology like AI in healthcare. They have to be responsible and make sure they do the right things. Keeping patients' information private is crucial, and they have to think about what is right and wrong in every decision they make about using AI. It's like a big change, and nurses have to think about how it can help patients, but also be careful about any problems it might cause." *(Code: R08, Female, Nursing incharge, 19 years of working experience)*

Participants in the qualitative research emphasized their conscientious approach to integrating AI in nursing, underscoring a deep understanding of the ethical implications. Their commitment to responsible AI usage is reflected in their dedication to thoughtful decision-making and adherence to established rules. The nurses prioritize maintaining trust by upholding promises, ensuring a secure and well-managed healthcare environment. This qualitative insight highlighted the crucial intersection of technology and patient care, demonstrating a collective commitment to ethical and responsible AI implementation within the nursing profession.

"As nurses, we understand the importance of using AI responsibly. Mixing technology and taking care of patients needs careful thinking. We make sure to use AI in the right way, keeping our promises. We promise to follow the rules and do our job responsibly so that the people who trust us for their health can feel safe and well taken care of." *(Code: R02, Male, Senior staff nurse, 4 years of working experience)*

### 4.3 Privacy challenges in healthcare AI

#### 4.3.1 Data security and confidentiality

In navigating the intricate landscape of AI, participants voiced a paramount concern: ensuring the safety and privacy of patient information (Table 3). As nurses, they identified themselves as guardians entrusted with patient confidence. Striving for responsibility, they emphasized the need to maintain privacy while integrating new technologies. Rigorous checks during digital interactions and computer program usage were highlighted to prevent any inadvertent sharing of confidential patient details. Beyond their protective role, participants viewed themselves as builders, contributing to a secure healthcare future where AI seamlessly aligns while safeguarding patient information—a parallel to the special connection they share with their patients.

"In the complicated world of using AI, our main worry is keeping patient information safe and private. As nurses, we're like the guardians of the trust patients put in us. We have to make sure that as we use new technology, we're also being responsible and keeping things private. Every time we use a computer program or connect digitally, we check to make sure we're not sharing anything about our patients that should be kept secret. We're not just protectors; we're like builders creating a safe healthcare future where AI fits in well while keeping patient information safe, just like the special connection we have with our patients." *(Code: R16, Female, Nursing manager, 27 years of working experience)*

#### 4.3.2 Algorithmic transparency and accountability

In this study, participants expressed a profound fusion of nursing care and technological expertise, emphasizing their dual roles as caregivers and guardians of digital confidentiality. Describing the intricate nature of handling “tricky computer stuff” and secrets embedded in numbers, participants highlighted the use of transparency as a guiding light to ensure ethical practices. In the realm of healthcare and technology support, nurses were metaphorically portrayed as robust protectors, dedicated to safeguarding individuals' private information from unauthorized access or misuse, embodying a unique blend of compassion and digital vigilance.

"Nurses take care of people, and now they also deal with tricky computer stuff - secrets hidden in numbers. They use transparency like a light to make sure they're doing the right thing. In the world of healthcare and computer help, nurses are like strong protectors, making sure no one's private information gets into the wrong hands." *(Code: R10, Male, Senior staff nurse, 21 years of working experience)*

#### 4.3.3 Patient advocacy for privacy

Nurses, envisioned as leaders in the healthcare drama where computers play a crucial role, were likened to central figures making solemn promises. Each facet of this intricate play represented a commitment to safeguard private information for those under their care. Participants emphasized the significant pledge to protect the unique trust shared between nurses and the individuals they assist. In this portrayal, nurses emerged as guardians, dedicated to upholding the sanctity of confidentiality, epitomizing a profound responsibility in the realm of healthcare intertwined with technology.

"Imagine nurses as the leaders in a play when computers help in healthcare. Each part of the play is like a promise to keep private things safe for the people they help. It's a big promise to protect the special trust between the nurse and the person they are helping." *(Code: R14, Female, Senior staff nurse, 9 years of working experience)*

### 4.4 Balancing innovation and ethical practice

#### 4.4.1 Technology-enhanced patient outcomes

Within this era of marvels, nurses emerged as key custodians, tasked with maintaining equilibrium between progress and ethical imperatives (Table 4). Acknowledging the transformative impact of AI on healthcare outcomes, participants underscored the paramount importance of ethical considerations. The poetic expression captured the essence of nurses' roles, navigating the dynamic landscape of technological advancements while steadfastly upholding ethical standards, highlighting the delicate balance required in the intersection of nursing care and the transformative touch of artificial intelligence.

"In the era of marvels, nurses hold the key,

To balance progress and ethical decree.

AI's transformative touch on outcomes profound,

Yet, our duty lies in ethical surroundings." *(Code: R03, Female, Nursing manager, 22 years of working experience)*

**Table 4:** Themes, sub-themes, and codes.

**Themes**	**Sub-themes**	**Codes**
Balancing innovation and ethical practice	Technology-enhanced patient outcomes	Era, Nurses, Key, Balance, Progress, Ethical decree, AI, Transformative touch, Outcomes, Duty, Ethical surroundings
	Ethical challenges in AI implementation	AI adoption, Healthcare, Change, Nurses, Ethical practice, Algorithms, Navigating, Patient rights, Confidentiality, Trust, Ethical challenges, Responsible future, Hurdles
	Patient empowerment through technology	Nursing, Artificial intelligence, Nuanced approach, Patient empowerment, Technology, Intersection, Innovation, Ethical practice, Cutting-edge tools, Patient voices, Sacred trust, Privacy, Digital tapestry, Balancing, Ethics, Champion, Click
Human touch vs. technological progress	Maintaining emotional connection	Technology, Nursing, Essence, Constantly connected, Patients, Human level, Duty, Warmth of empathy, Cold hum of machinery
	Patient perception of AI in care	Patients, Technology, Detachment, AI, Machines, Seamless blend, Technological prowess, Compassionate caregiving

#### 4.4.2 Ethical challenges in AI implementation

The findings of this study emphasized the transformative impact of AI adoption in healthcare, as nurses highlighted a paradigm shift. They stressed the pivotal role of ethical practice, emphasizing that beyond algorithmic implementation lies a complex landscape of patient rights, confidentiality, and trust. Acknowledging the challenges, nurses recognized the imperative of navigating these ethical hurdles for a responsible AI-infused future, emphasizing the significance of upholding ethical standards amidst the evolving healthcare landscape.

"AI adoption in healthcare brings a wave of change, but as nurses, our compass is ethical practice. It's not just about implementing algorithms; it's about navigating the intricate terrain of patient rights, confidentiality, and trust. The ethical challenges in AI are the hurdles we must clear for a responsible future." *(Code: R11, Female, Nursing incharge, 16 years of working experience)*

#### 4.4.3 Patient empowerment through technology

In this qualitative research, nurses articulated a nuanced perspective on nursing in the age of artificial intelligence. The findings highlighted a commitment to patient empowerment through technology, positioning it as a guiding principle. Nurses emphasized their role at the intersection of innovation and ethical practice, navigating a path where cutting-edge tools amplify patient voices while safeguarding the sacred trust embedded in privacy. These insights underscored the delicate balance sought in harnessing AI's potential to enhance healthcare without compromising the fundamental values of compassionate and confidential patient care.

"Nursing in the age of artificial intelligence demands a nuanced approach. Patient empowerment through technology is our guidepost. We are at the intersection of innovation and ethical practice, carving a path where cutting-edge tools amplify patient voices without compromising the sacred trust embedded in privacy." *(Code: R07, Female, Senior staff nurse, 11 years of working experience)*

In this study, nurses expressed their commitment to patient empowerment within the digital healthcare landscape. Their quotes underscored the delicate balance between embracing innovation and upholding ethical standards. The nurses passionately emphasized their role as advocates for patient privacy, weaving it seamlessly into the fabric of every digital interaction, thereby fostering a compassionate and secure healthcare environment.

"We're nurses, weaving patient empowerment into the digital tapestry. Balancing innovation with ethics, we champion privacy with every click." *(Code: R01, Female, Nursing incharge, 15 years of working experience)*

### 4.5 Human touch vs. technological progress

#### 4.5.1 Maintaining emotional connection

The findings of this study emphasized the enduring human connection at the heart of nursing, regardless of evolving technology (Table 4). Nurses conveyed a commitment to maintaining a profound connection with patients on a personal level. The nurses highlighted their duty to infuse empathy into the clinical environment, ensuring that amidst the impersonal hum of machinery, every patient experiences the enduring warmth of compassionate care.

"The technology may change, but the essence of nursing remains constantly connected with patients on a human level. I believe our duty is to ensure that every patient feels the warmth of empathy amidst the cold hum of machinery." *(Code: R09, Female, Nursing manager, 25 years of working experience)*

#### 4.5.2 Patient perception of AI in care

Nurses shared insights on patient perspectives toward technology. They emphasized that patients' concerns lie not in technology itself but in the potential for detachment. Nurses see their role as bridging this gap, advocating for a seamless integration of AI while preserving compassionate caregiving. The focus is on transforming the patient's experience to perceive not just machines but a harmonious fusion of technological expertise and empathetic care.

"Patients don't fear technology; they fear detachment. Our task is to ensure that in the era of AI, they don't just see machines but witness a seamless blend of technological prowess and compassionate caregiving." *(Code: R20, Male, Senior staff nurse, 7 years of working experience)*

### 4.6 Patient-centered care in the AI era

#### 4.6.1 Customization of care plans

In this research, nurses highlighted the evolution of patient-centered care in the age of AI (Table 5). They described it as a harmonious orchestration of personalized solutions, emphasizing that true compassion lies in customization. The nurses expressed a commitment to tailoring every aspect of the care journey to meet the unique needs and preferences of each individual in their care. This approach ensures a more empathetic and responsive healthcare experience, fostering a deeper connection between caregivers and patients in the era of advanced technology.

"Patient-centered care in the AI era is an orchestration of personalized solutions. I believe customization is at the core of compassion, tailoring each step of the care journey to the specific needs and preferences of the individual in our care." *(Code: R04, Female, Nursing incharge, 17 years of working experience)*

**Table 5:** Themes, sub-themes, and codes.

**Themes**	**Sub-themes**	**Codes**
Patient-centered care in the AI era	Customization of care plans	Patient-centered care, AI era, Orchestration, Personalized solutions, Customization, Compassion, Care journey, Specific needs, Preferences, Individual care
	Maintaining patient-centered communication	Communication, Bridge, Patients, Heart of care Challenge, Interaction, Empathy, Understanding Commitment, Well-being, Patient-centered communication, Lifeline, Dialogue, Empowerment, Comfort, Human touch
Ethical preparedness and education	Training programs on ethical AI use	Advancing technology, Ethical preparedness, Patient care, Training programs, Ethical AI use, Compass, Complexities, Interaction, Artificial intelligence, Commitment, Moral principles, Patient management
	Interdisciplinary collaboration in ethics	AI, Healthcare, Collaboration, Technology, Ethical understanding, Commitment, Prioritize, Fairness, Goodness, Success, Right, Everyone involved, Helper, Change, Collaboration
	Organizational support for ethical decision-making	Ethical decision-making, Collective responsibility, Organizational support, AI-integrated clinical setting, Policies, Culture, Ethical mindfulness, Collective responsibility, Organizational support, Ethical decision-making

#### 4.6.2 Maintaining patient-centered communication

Nurses stressed the pivotal role of communication as the bridge to the essence of care (Table 5). They acknowledged the challenge of infusing every interaction with empathy, understanding, and an authentic dedication to the patient's well-being. The quote underscored the nurses' awareness of the profound impact effective communication holds in cultivating a therapeutic relationship, emphasizing the need for continuous efforts to enhance the quality of interactions for the holistic well-being of patients under their care.

"I am certain that communication serves as the bridge connecting patients to the heart of care. However, our challenge is to ensure that every interaction reflects empathy, understanding, and a genuine commitment to the patient's well-being." *(Code: R12, Female, Senior staff nurse, 3 years of working experience)*

In our study, nurses emphasized the pivotal role of patient-centered communication, considering it the lifeline of care. They stressed its ability to establish a dialogue that empowers and comforts the patient. The nurses expressed a commitment to ensuring that their conversations are as human as the tactile care they provide, underscoring the importance of genuine and compassionate communication in delivering holistic and patient-focused healthcare experiences.

"Patient-centered communication is the lifeline of care. It creates a dialogue that empowers and comforts the patient. Consequently, our conversations should be as human as the touch we offer." *(Code: R18, Male, Senior staff nurse, 8 years of working experience)*

### 4.7 Ethical preparedness and education

#### 4.7.1 Training programs on ethical AI use

**Fig. 2:**
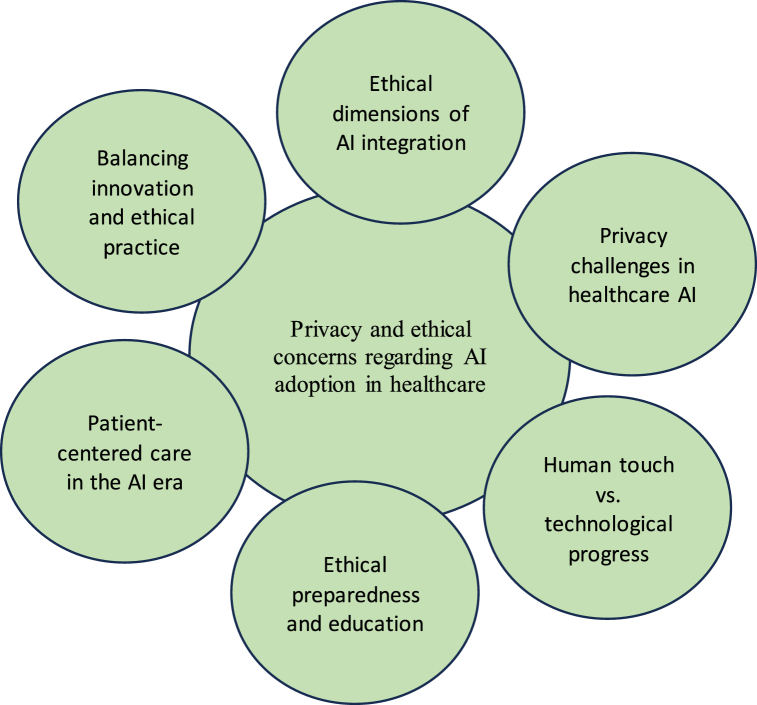
Depicts the thematic analysis of nurses' perspectives on privacy and ethical concerns regarding AI adoption in healthcare.

In this qualitative research, nurses emphasized the importance of ethical preparedness in the rapidly advancing era of technology (Fig. 2). They viewed training programs on ethical AI use as indispensable, acting as a compass through complexities in patient care. The quote highlighted a collective commitment among nurses to ensure that every interaction with artificial intelligence aligns with moral principles, underscoring the significance of ethical considerations in navigating the integration of technology within the realm of patient management.

"In the era of advancing technology, ethical preparedness is crucial for patient care. Training programs on ethical AI use act as the compass guiding us through complexities, ensuring that every interaction with artificial intelligence reflects our commitment to moral principles in patient management." *(Code: R15, Female, Nursing incharge, 14 years of working experience)*

#### 4.7.2 Interdisciplinary collaboration in ethics

In this study, nurses envisaged AI as a transformative healthcare ally, revolutionizing operational processes. They stressed the imperative for collective collaboration, uniting individuals with expertise in healthcare, technology, and ethics. This interdisciplinary alliance was recognized as the cornerstone for establishing an unwavering commitment to prioritizing fairness and ethical principles. In the realm of healthcare integrated with AI, success goes beyond technological efficiency; it signifies a steadfast dedication to consistently doing what is ethically right for all stakeholders immersed in the intricate landscape of patient care.

"Imagine AI as a helper in healthcare, changing how things work. To ensure that AI can be used in the right way, everyone needs to work together. People who understand healthcare, technology, and have ethical understanding join forces. This collaboration will build a strong commitment to always prioritize fairness and goodness. In a healthcare setting with AI, success does not only mean the technology working well; it means ensuring that we always do what is right for everyone involved." *(Code: R06, Male, Senior staff nurse, 6 years of working experience)*

#### 4.7.3 Organizational support for ethical decision-making

The findings of this study underscored the collective responsibility of ethical decision-making. Nurses emphasized the crucial need for organizational support in every decision, particularly within the AI-integrated clinical setting. Merely formulating policies proved insufficient; instead, there was an imperative to cultivate a culture of ethical mindfulness. The quote revealed a call for comprehensive measures beyond mere guidelines, urging organizations to actively nurture an environment where ethical considerations are woven into the fabric of decision-making processes, ensuring the alignment of AI practices with moral principles in healthcare.

"From my sense ….ethical decision-making is a collective responsibility. Organizational support needs to be ensured for every decision, especially in the AI-integrated clinical setting. Simply making policies is not enough; there is a need to foster a culture of ethical mindfulness." *(Code: R17, Male, Senior staff nurse, 5 years of working experience)*

Fig. 2 depicts the thematic analysis of nurses' perspectives on privacy and ethical concerns regarding AI adoption in healthcare.

## 5 Discussion

This study explored nurses' perspectives on privacy and ethical concerns associated with the implementation of AI in healthcare settings. Nurses stand at the forefront of healthcare delivery, and their perspectives on the integration of artificial intelligence (AI) into healthcare systems are instrumental in shaping ethical practices and safeguarding patient privacy [56]. As the healthcare landscape continues to evolve with technological advancements, understanding nurses' viewpoints on privacy and ethical concerns regarding AI adoption is critical for promoting patient-centered care while upholding ethical standards [57]. This study revealed that ethical dimensions of AI integration represent a fundamental aspect of nurses' considerations in adopting AI technologies in healthcare settings. Informed decision-making emerges as a cornerstone principle, wherein nurses prioritize transparent communication with patients regarding AI-driven interventions. “Imagine nurses as the leaders in a play when computers help in healthcare. Each part of the play is like a promise to keep private things safe for the people they help. It's a big promise to protect the special trust between the nurse and the person they are helping,” said one nurse, emphasizing the pivotal role nurses play in maintaining patient privacy and trust. A study by Han et al. (2022) underscored the significance of patient engagement and shared decision-making in AI-enabled care delivery [58]. Similarly, Liaw et al. (2023) emphasized the need for healthcare providers, including nurses, to facilitate open dialogue with patients to ensure their informed participation in AI-driven healthcare decisions [59]. By involving patients in the decision-making process, nurses uphold ethical principles of autonomy and respect for patient preferences [60].

This research found that ethical guidelines and frameworks serve as essential tools for guiding nurses in navigating the complexities of AI integration in healthcare. Fujimori et al. (2022) highlighted the role of established ethical principles in shaping AI development and deployment [61]. Nurses advocate for adherence to ethical guidelines to ensure that AI systems prioritize patient welfare and uphold ethical standards [62]. Furthermore, Jordan et al. (2023) emphasized nurses' professional accountability and responsibility in monitoring AI algorithms for biases and ensuring equitable care delivery [63]. By adhering to ethical frameworks and standards, nurses contribute to the ethical integrity of AI adoption in healthcare [64].

In this study, nurses expressed that privacy challenges in healthcare AI present significant concerns for nurses, particularly regarding data security and confidentiality. Hacking et al. (2022) highlighted nurses' apprehensions regarding the protection of sensitive patient information in AI systems [65]. Studies underscored the pressing need for robust data encryption protocols and stringent privacy measures to safeguard patient confidentiality [66,67]. Moreover, algorithmic transparency and accountability emerge as focal points in addressing privacy challenges [68]. Laukka et al. (2022) emphasized the importance of comprehensible AI algorithms that enable healthcare providers, including nurses, to understand decision-making processes [69]. By ensuring transparency and accountability in AI algorithms, nurses mitigate privacy risks and uphold patient confidentiality [70].

This study revealed that patient advocacy for privacy represents a crucial aspect of nurses' roles in AI-driven healthcare. Hwang et al. (2022) highlighted nurses' efforts in advocating for patient privacy rights amidst rapid technological advancements [71], and research by Kitsios et al. (2023) mentioned the significance of this advocacy [72]. Nurses play a vital role in ensuring that AI technologies prioritize patient confidentiality and respect patient autonomy [73]. “Patients don't fear technology; they fear detachment. Our task is to ensure that in the era of AI, they don't just see machines but witness a seamless blend of technological prowess and compassionate caregiving,” added a nurse, emphasizing the importance of human connection in healthcare. By advocating for privacy protection, nurses uphold ethical principles of patient-centered care and promote trust between patients and healthcare providers [74].

This research identified that balancing innovation with ethical practice poses challenges for nurses in the adoption of AI technologies. While technology-enhanced patient outcomes hold promise for improving healthcare delivery, nurses remain vigilant about the ethical implications of AI implementation. Cheng et al. (2023) underscored nurses' role in navigating ethical dilemmas inherent in AI-driven care delivery, emphasizing the importance of maintaining a patient-centered approach [75]. Patient empowerment through technology emerges as a promising avenue for enhancing patient engagement and autonomy in healthcare decision-making [76]. Barwise et al. (2024) highlighted nurses' efforts to leverage AI tools to empower patients in managing their health effectively [77]. By providing patients with access to personalized health information and decision support, nurses facilitate informed choices and promote patient-centered care [78].

However, nurses also grapple with the challenge of maintaining the human touch amidst technological progress. Kaduwela et al. (2024) underscored nurses' concerns about preserving emotional connections with patients in an increasingly digitized healthcare environment [79]. In Despite the potential benefits of AI in streamlining processes and improving efficiency, nurses emphasized the irreplaceable value of human compassion and empathy in patient care. Patient perception of AI in care represents another critical area of inquiry for nurses. Kannelønning (2024) highlighted the importance of addressing patient concerns and misconceptions about AI technologies to foster trust and acceptance [80]. Nurses play a vital role in educating patients about the benefits and limitations of AI in healthcare, facilitating a more positive perception and adoption of these technologies [81].

This study also highlighted that patient-centered care remains paramount in the AI era, with nurses advocating for the customization of care plans tailored to individual patient needs and preferences. Rony et al. (2024) emphasized the importance of incorporating patient input and feedback into AI-driven care models, ensuring that technology complements rather than supplants human-centered care approaches [34], while recent review by Moy et al. (2024) further underscored the significance of this patient-centered approach [82]. Maintaining patient-centered communication is essential for fostering trust and collaboration between nurses and patients [21]. By prioritizing open communication and patient engagement, nurses promote patient-centered care delivery in the AI era [6].

In this research, participants explained that ethical preparedness and education are essential for equipping nurses with the knowledge and skills to navigate the ethical complexities of AI integration in healthcare. Training programs on ethical AI use provide nurses with the necessary tools to uphold ethical standards in their practice. Kwak et al. (2022) highlighted the importance of interdisciplinary collaboration in ethics, fostering a holistic approach to addressing ethical challenges in AI-driven healthcare [31]. Moreover, organizational support for ethical decision-making is crucial in creating a culture of ethical practice in healthcare settings [18]. Ahmed, (2024) underscored the importance of organizational policies and resources that prioritize ethical considerations in AI implementation [83]. By providing nurses with the support they need to navigate ethical dilemmas, healthcare organizations can ensure that AI adoption enhances patient care outcomes while upholding ethical integrity [84].

## 6 Implications of the study

This study, which investigates nurses' perspectives on privacy and ethical concerns regarding the adoption of artificial intelligence (AI) in healthcare, reveals crucial implications for policymakers, healthcare institutions, and AI developers. Firstly, it underscores the pressing need for comprehensive guidelines and regulations governing the integration of AI technologies in healthcare settings to address privacy breaches and ethical dilemmas. Nurses' apprehensions regarding patient data security and confidentiality highlight the importance of implementing robust data protection measures and ensuring transparent AI algorithms. Moreover, the findings emphasize the significance of providing adequate training and education to nurses regarding AI systems to alleviate their ethical concerns and enhance their confidence in utilizing these technologies effectively. Healthcare organizations must invest in continuous professional development programs to empower nurses with the knowledge and skills necessary to navigate the ethical complexities associated with AI adoption. From an international perspective, this paper contributes by shedding light on how nurses across different healthcare systems perceive the ethical and privacy challenges posed by AI integration, offering valuable insights for global policy and practice considerations.

Additionally, the study underscores the importance of fostering interdisciplinary collaboration between nurses, technologists, ethicists, and policymakers to develop AI solutions that prioritize patient privacy, autonomy, and beneficence. By engaging in multi-stakeholder discussions and incorporating diverse perspectives, healthcare providers can promote the responsible and ethical deployment of AI technologies while safeguarding patient rights and interests. Furthermore, the study highlights the need for ongoing monitoring and evaluation of AI systems' impact on patient privacy and ethical standards to identify and address emerging challenges proactively. Continuous assessment and refinement of AI algorithms and protocols are essential to ensure alignment with ethical principles and regulatory requirements while promoting patient-centered care delivery. This study is increasing our understanding of how interdisciplinary collaboration can enhance the ethical deployment of AI in healthcare, thereby facilitating the development of more patient-centered and ethically sound practices. Overall, this study underscores the imperative of integrating ethical considerations and privacy safeguards into the development and implementation of AI technologies in healthcare to uphold professional values, protect patient rights, and foster trust in healthcare delivery systems.

## 7 Strengths and limitations

The strength of this study lies in its robust methodological approach and adherence to qualitative research standards, ensuring credibility, transparency, and rigor in exploring nurses' perspectives on privacy and ethical concerns regarding AI adoption in healthcare. By employing Van Manen's hermeneutic phenomenology, the research delved deeply into the lived experiences and interpretive understanding of nurses, providing rich insights into the subjective dimensions of the phenomena under investigation. Moreover, the study adhered to the COREQ checklist, ensuring comprehensive reporting of study design, analysis, and findings, thereby enhancing the transparency and reproducibility of the research process.

However, several limitations should be considered when interpreting the results. Firstly, the study's focus on nurses' perspectives may overlook the viewpoints of other healthcare stakeholders, such as patients, physicians, and AI developers, potentially limiting the comprehensiveness of the findings. Additionally, the use of purposive sampling from four tertiary hospitals in Dhaka city, Bangladesh, may restrict the generalizability of the results to broader populations of nurses working in different healthcare settings or geographic locations. The inclusion criteria specifying nurses with a minimum master's degree may introduce selection bias, potentially excluding perspectives from nurses with different educational backgrounds. Furthermore, the translation of data from Bangla to English may result in the loss of nuance or interpretation errors, affecting the accuracy and reliability of data analysis and interpretation. Considering theoretical limitations, such as the absence of a specific theoretical framework guiding the research, may also impact the interpretation of findings and limit the depth of theoretical insights derived from the study. Despite these limitations, the study provides valuable insights into nurses' perceptions of privacy and ethical considerations surrounding AI adoption in healthcare, serving as a foundation for further research in this evolving field.

## 8 Recommendations for future research

Future research in the realm of healthcare AI should prioritize several key areas based on our findings. Firstly, there is a pressing need to delve deeper into the ethical dimensions of AI integration, recognizing the complexities inherent in incorporating AI ethically into healthcare systems. This includes developing comprehensive frameworks that address the ethical challenges posed by AI use while safeguarding patient welfare. Secondly, addressing privacy challenges in healthcare AI is paramount, with research focusing on strategies to ensure data security and confidentiality amidst increasing digitalization. Thirdly, efforts should be directed towards reconciling innovation with ethical practice, exploring how to navigate the tension between technological advancements and ethical considerations. Additionally, studies should investigate the interplay between human touch and technological progress, examining how automation impacts personalized care delivery. Moreover, maintaining a steadfast commitment to patient-centered care in the AI era requires ongoing research to ensure that technological advancements do not detract from focusing on patients' needs and preferences. Lastly, enhancing ethical preparedness and education among healthcare professionals is essential, with research focusing on the development and evaluation of training programs that equip practitioners with the necessary skills to navigate ethical dilemmas arising from AI use. By prioritizing these areas, future research endeavors can contribute to the responsible and ethical integration of AI in healthcare practice.

## 9 Conclusions

The perspectives of nurses regarding privacy and ethical concerns surrounding the integration of artificial intelligence (AI) in healthcare reveal multifaceted considerations. Nurses, as frontline healthcare providers, exhibit a nuanced understanding of the potential benefits and pitfalls of AI adoption. While acknowledging the promise of AI in enhancing efficiency and patient care, they express apprehensions about data privacy, patient confidentiality, and the ethical implications of AI decision-making. These concerns underscore the importance of ensuring robust privacy protocols, transparent AI algorithms, and ongoing ethical oversight in AI implementation within healthcare settings. Moreover, nurses emphasize the significance of maintaining human-centric care, where AI complements rather than replaces the vital role of human judgment and empathy. As healthcare continues to evolve with technological advancements, collaborative efforts involving nurses, policymakers, ethicists, and technologists are imperative to navigate the complex landscape of AI adoption while upholding ethical principles and safeguarding patient privacy.

## CRediT authorship contribution statement

**Moustaq Karim Khan Rony:** Writing – review & editing, Writing – original draft, Visualization, Validation, Supervision, Software, Resources, Project administration, Methodology, Investigation, Formal analysis, Data curation, Conceptualization. **Sharker Md. Numan:** Writing – review & editing, Software, Methodology, Formal analysis. **Khadiza Akter:** Writing – original draft, Visualization, Validation, Data curation. **Hasanuzzaman Tushar:** Writing – original draft, Project administration, Methodology, Formal analysis. **Mitun Debnath:** Validation, Project administration, Investigation, Data curation. **Fateha tuj Johra:** Visualization, Resources, Project administration, Formal analysis. **Fazila Akter:** Writing – review & editing, Visualization, Resources, Formal analysis. **Sujit Mondal:** Resources, Project administration, Methodology, Investigation. **Mousumi Das:** Writing – review & editing, Software, Data curation. **Muhammad Join Uddin:** Writing – review & editing, Project administration, Investigation, Data curation. **Jeni Begum:** Visualization, Supervision, Conceptualization. **Mst. Rina Parvin:** Writing – review & editing, Writing – original draft, Validation, Supervision, Project administration, Methodology, Formal analysis, Data curation, Conceptualization.

## Declaration of competing interest

The authors declare that they have no known competing financial interests or personal relationships that could have appeared to influence the work reported in this paper.
