# Supplementary material for: Nurses' perspectives on privacy and ethical concerns regarding artificial intelligence adoption in healthcare
Source: Heliyon. 2024 Aug 22;10(17):e36702. doi: 10.1016/j.heliyon.2024.e36702 (PMC11400963; doi:10.1016/j.heliyon.2024.e36702)
Supplement: Multimedia component 1 [file mmc1.docx]

"We're nurses, weaving patient empowerment into the digital tapestry. Balancing innovation with ethics, we champion privacy with every click." ***(Code: R01, Female, Nursing incharge, 15 years of working experience)***

"As nurses, we understand the importance of using AI responsibly. Mixing technology and taking care of patients needs careful thinking. We make sure to use AI in the right way, keeping our promises. We promise to follow the rules and do our job responsibly so that the people who trust us for their health can feel safe and well taken care of." ***(Code: R02, Male, Senior staff nurse, 4 years of working experience)***

"In the era of marvels, nurses hold the key,

To balance progress and ethical decree.

AI's transformative touch on outcomes profound,

Yet, our duty lies in ethical surroundings." ***(Code: R03, Female, Nursing manager, 22 years of working experience)***

"Patient-centered care in the AI era is an orchestration of personalized solutions. I believe customization is at the core of compassion, tailoring each step of the care journey to the specific needs and preferences of the individual in our care." ***(Code: R04, Female, Nursing incharge, 17 years of working experience)***

"Nurses are like caring protectors in healthcare. They follow the rules and do what's right, especially when new things like AI are being used. We promise to keep patients' information private and treat them with respect. Even in a world of technology and information, we make sure that progress doesn't forget the important values of our job. Ethical rules are like walls that keep the trust between nurses and the people we take care of safe." ***(Code: R05, Male, Senior staff nurse, 13 years of working experience)***

"Imagine AI as a helper in healthcare, changing how things work. To ensure that AI can be used in the right way, everyone needs to work together. People who understand healthcare, technology, and have ethical understanding join forces. This collaboration will build a strong commitment to always prioritize fairness and goodness. In a healthcare setting with AI, success does not only mean the technology working well; it means ensuring that we always do what is right for everyone involved." ***(Code: R06, Male, Senior staff nurse, 6 years of working experience)***

"Nursing in the age of artificial intelligence demands a nuanced approach. Patient empowerment through technology is our guidepost. We are at the intersection of innovation and ethical practice, carving a path where cutting-edge tools amplify patient voices without compromising the sacred trust embedded in privacy." ***(Code: R07, Female, Senior staff nurse, 11 years of working experience)***

"Nurses are very important when it comes to using new technology like AI in healthcare. They have to be responsible and make sure they do the right things. Keeping patients' information private is crucial, and they have to think about what is right and wrong in every decision they make about using AI. It's like a big change, and nurses have to think about how it can help patients, but also be careful about any problems it might cause." ***(Code: R08, Female, Nursing incharge, 19 years of working experience)***

"The technology may change, but the essence of nursing remains constantly connected with patients on a human level. I believe our duty is to ensure that every patient feels the warmth of empathy amidst the cold hum of machinery." ***(Code: R09, Female, Nursing manager, 25 years of working experience)***

"Nurses take care of people, and now they also deal with tricky computer stuff - secrets hidden in numbers. They use transparency like a light to make sure they're doing the right thing. In the world of healthcare and computer help, nurses are like strong protectors, making sure no one's private information gets into the wrong hands." ***(Code: R10, Male, Senior staff nurse, 21 years of working experience)***

"AI adoption in healthcare brings a wave of change, but as nurses, our compass is ethical practice. It's not just about implementing algorithms; it's about navigating the intricate terrain of patient rights, confidentiality, and trust. The ethical challenges in AI are the hurdles we must clear for a responsible future." ***(Code: R11, Female, Nursing incharge, 16 years of working experience)***

"I am certain that communication serves as the bridge connecting patients to the heart of care. However, our challenge is to ensure that every interaction reflects empathy, understanding, and a genuine commitment to the patient's well-being." ***(Code: R12, Female, Senior staff nurse, 3 years of working experience)***

"When we use AI to help take care of patients, it's not just pressing buttons; it's like creating a story with our choices. Each decision is like a part of a story, not just finishing tasks. We look at a lot of information, making sure our choices aren't quick but a well-thought-out plan, following what's right. Making good decisions with AI in patient care is like telling a story where the ideas come from thinking carefully, making a tale of choices that follow the rules we believe in." ***(Code: R13, Male, Senior staff nurse, 7 years of working experience)***

"Imagine nurses as the leaders in a play when computers help in healthcare. Each part of the play is like a promise to keep private things safe for the people they help. It's a big promise to protect the special trust between the nurse and the person they are helping." ***(Code: R14, Female, Senior staff nurse, 9 years of working experience)***

"In the era of advancing technology, ethical preparedness is crucial for patient care. Training programs on ethical AI use act as the compass guiding us through complexities, ensuring that every interaction with artificial intelligence reflects our commitment to moral principles in patient management." ***(Code: R15, Female, Nursing incharge, 14 years of working experience)***

"In the complicated world of using AI, our main worry is keeping patient information safe and private. As nurses, we're like the guardians of the trust patients put in us. We have to make sure that as we use new technology, we're also being responsible and keeping things private. Every time we use a computer program or connect digitally, we check to make sure we're not sharing anything about our patients that should be kept secret. We're not just protectors; we're like builders creating a safe healthcare future where AI fits in well while keeping patient information safe, just like the special connection we have with our patients." ***(Code: R16, Female, Nursing manager, 27 years of working experience)***

"From my sense......ethical decision-making is a collective responsibility. Organizational support needs to be ensured for every decision, especially in the AI-integrated clinical setting. Simply making policies is not enough; there is a need to foster a culture of ethical mindfulness." ***(Code: R17, Male, Senior staff nurse, 5 years of working experience)***

"Patient-centered communication is the lifeline of care. It creates a dialogue that empowers and comforts the patient. Consequently, our conversations should be as human as the touch we offer." ***(Code: R18, Male, Senior staff nurse, 8 years of working experience)***

"I believe that as nurses using AI in healthcare, it's important to follow ethical guidelines. When we use new technology, we don't forget about what's right and wrong; instead, we make our sense of what's right even stronger. Ethical rules aren't rules that limit us; they're like bright signs showing us the way to keep our journey honest, caring, and true to the principles of our job. As we move forward with new ideas and ethics, we hold the responsibility of using AI in a way that respects patient privacy and follows the values that make healthcare special." ***(Code: R19, Female, Nursing incharge, 15 years of working experience)***

"Patients don't fear technology; they fear detachment. Our task is to ensure that in the era of AI, they don't just see machines but witness a seamless blend of technological prowess and compassionate caregiving." ***(Code: R20, Male, Senior staff nurse, 7 years of working experience)***
